# JOBS Program Germany for health promotion among the unemployed in the community setting with institutions for employment promotion (JobsProgramDtl): study protocol for a randomized controlled trial

**DOI:** 10.1186/s12889-021-10251-8

**Published:** 2021-02-01

**Authors:** Alfons Hollederer, Heiko J. Jahn, Daniel Klein

**Affiliations:** The Faculty of Human Sciences (FB 01), Department of Social Work and Social Welfare, Chair for Theory and Empirics of Health, University of Kassel, Arnold-Bode-Str. 10, D-34109 Kassel, Germany

**Keywords:** Unemployment, Health, Health promotion, Prevention, JOBS Program, Germany

## Abstract

**Background:**

Compared to the employed, the unemployed are characterized by a substantially worse health status, particularly with regards to mental health. At the same time, conventional offers of prevention and health promotion rarely reach the unemployed. The JOBS Program is a health promotion program that combines job application training with elements of social learning theory and self-efficacy. Randomized field studies in the USA and Finland found significant positive effects on reintegration into the labor market and health amongst the unemployed. In this confirmatory study, we analyze whether the JOBS Program produces similar positive effects for the unemployed in Germany.

**Methods:**

This study is designed as a country-wide, multi-center, non-blinded, two-armed, parallel-group, randomized controlled trial over 6 months. A total of approximately 1500 unemployed, who are willing to participate, are randomly assigned either to an intervention group or a waiting control group with an allocation ratio of 1:1. Guided by a team of two trainers, the intervention group completes JOBS Program Germany in small groups of 8 to 15 unemployed for a period of 1 week in units of 5 h a day. Primary outcome measures are the reintegration into the labor market, self-efficacy, life satisfaction, subjective state of health, depressive symptoms, and psychological distress. Of secondary interest are moderating variables such as socio-demographic characteristics, the duration of unemployment, and the job-search intensity. Outcomes will be repeatedly assessed via computer-assisted telephone interviews at three points in time: immediately before the intervention (pre-test), immediately after the intervention (post-test), and 6 months after the intervention has ended (6-months follow-up).

**Discussion:**

This confirmatory study will provide empirical evidence on the effectiveness of the JOBS Program on the reintegration and (mental) health of the unemployed in Germany. If our results from the randomized controlled trail in a country-wide field experiment confirm its effectiveness, the JOBS Program Germany could, perspectively, be implemented into the German employment promotion and social security system on a long-term basis.

**Trial registration:**

German Clinical Trials Register (DRKS), DRKS00022388. Registered on 20 July, 2020.

## Background

Unemployment poses a problem for society as a whole and, in particular, a challenge for prevention and health promotion programs. Meta-analyses have shown that compared to the employed, the unemployed have a worse health status [1, 2], particularly with regards to mental health. At the same time, these studies suggest that reemployment fosters mental health. Conversely, poor health decreases the chances of reemployment [3]. Given these interactions between health and unemployment, health promotion is especially important for the unemployed. It is therefore unfortunate that conventional offers of prevention and health promotion rarely reach the unemployed [4].

A promising strategy to meet the challenges of improving the health among the unemployed is interdepartmental cooperation between health promotion and employment promotion. In Germany, the Federal Centre for Health Education (BZgA) and the National Association of Statutory Health Insurance Funds (GKV-Spitzenverband) are currently cooperating with the Federal Employment Agency on a large-scale program called “Linking of Employment Promotion and Health Promotion in the Community Setting”. Within this activity, they have adopted the so-called JOBS Program, which is a preventive intervention program that was originally developed in the 1990s by the Michigan Prevention Research Center [5–7]. The JOBS Program underpins elements of classical job application training with a socio-psychological theoretical foundation of social learning and self-efficacy [8, 9], thus, aiming at both increased employability and preventing negative consequences of unemployment on mental health. At its core, the JOBS Program is a training session (usually 1 week, 5 h a day) that is organized as a workshop in which small groups of unemployed develop job-search skills through active teaching and learning methods. Participants discuss potential setbacks (e.g., application rejections) and develop corresponding coping-strategies while trainers continuously provide additional social support.

Randomized field studies that have evaluated the JOBS Program in the United States [7, 10] and Finland [11] found significant positive effects on reintegration into the labor market and improved health status, such as lower levels of depressive symptoms and psychological distress, among the unemployed who had completed JOBS Program. These positive effects were observed repeatedly in a 6-months follow-up as well as 2 years after the program had ended [12, 13]. Similar results, especially with respect to employment, have been reported for a variation of the JOBS Program in Ireland [14]. A systematic review provides further information on how health promotion for the unemployed has been approached and evaluated [15].

While the positive results from the US, Finland, and Ireland seem promising, it remains an open empirical question whether the JOBS Program will unfold similar effects for the unemployed in Germany. From an international comparative perspective, it should be noted that the success of employment promotion and health promotion among the unemployed probably depends on the country-specific labor market structure and the health and social security systems, respectively. Also, the original JOBS Program was conceived as a preventive measure, primarily for the short-term unemployed, who have recently lost their jobs. In the US study, participants were unemployed for less than 13 weeks; the mean duration of employment in the Finnish study was about 10.7 months and only 28% of the participants had been unemployed for longer than 12 months. In Germany, the proportion of long-term unemployed is particularly high in international comparison. In 2019 about 32% have been unemployed for at least 1 year or longer [16]. Also, the comparatively low levels of education among the unemployed in Germany poses an additional obstacle for reintegration into the labor market.

A randomized control trial in the Netherlands [17] suggests that the positive effects of JOBS Program on the reemployment also apply to the long-term unemployed. More than half of the participants (54%) in the respective study had been unemployed for more than 5 years. The results confirm that JOBS Program fosters reemployment after 12 months among the participants in the intervention group. However, the study did not include measures of physical or mental health.

Against this background, our confirmatory study analyzes whether the JOBS Program increases reemployment and improves health among the unemployed in Germany.

### Specific objectives

The objective of this study is to investigate the effects of the JOBS Program intervention on the reintegration into the labor market, life satisfaction, state of health, depressive symptoms, and psychological distress among the unemployed in Germany. Of secondary interest are moderating factors such as socio-demographic characteristics, the duration of unemployment, and the job-search intensity.

We will compare the reintegration into the labor market and mental health amongst unemployed, who will be randomly assigned to an intervention group that completes JOBS Program Germany, to a waiting control group. The waiting control group will complete JOBS Program Germany only after the trial has terminated.

Given the confirmatory nature of this study, our hypotheses closely resemble those formulated in the respective studies that have investigated the JOBS Program in the US and Finland [10–13]. Compared to the waiting control group, the intervention group is expected to be more often and better reintegrated into the labor market. Moreover, the intervention group is also expected to report higher levels of life satisfaction, to assess its health status more favorably, and to have lower levels of psychological distress and depressive symptoms. More specifically, the following six hypotheses will be tested:

*Hypothesis1*: The JOBS Program Germany will increase the level and quality of reemployment in the intervention group compared to the waiting control group.

*Hypothesis 2*: The JOBS Program Germany will increase self-esteem and self-efficacy and decrease depressive symptoms and distress in the intervention group compared to the waiting control group.

*Hypothesis 3*: The risk of depression, assessed by a high level of depressive symptoms before the JOBS Program Germany, will moderate the effects of the JOBS Program Germany on reemployment and mental health outcomes. The JOBS Program Germany will have stronger employment promoting and mental health effects on those with a higher risk of depression.

*Hypothesis 4*: The job-search intensity, assessed before the JOBS Program Germany starts, will moderate the effects of the JOBS Program Germany on reemployment. The JOBS Program Germany will have stronger employment promoting effects on those with lower initial levels of job-search intensity.

*Hypothesis 5*: The length of unemployment will moderate the effects of the JOBS Program Germany on reemployment. The JOBS Program Germany will have a stronger employment promoting effect for those who have been unemployed for a moderate period compared to (1) the recently unemployed and (2) the long-term unemployed.

*Hypothesis 6*: The length of unemployment will moderate the effects of the JOBS Program Germany on mental health. The JOBS Program Germany will have stronger mental health effects for those who have been unemployed for a moderate period compared to (1) the recently unemployed and (2) the long-term unemployed.

## Methods

### Trial design

**Fig. 1:**
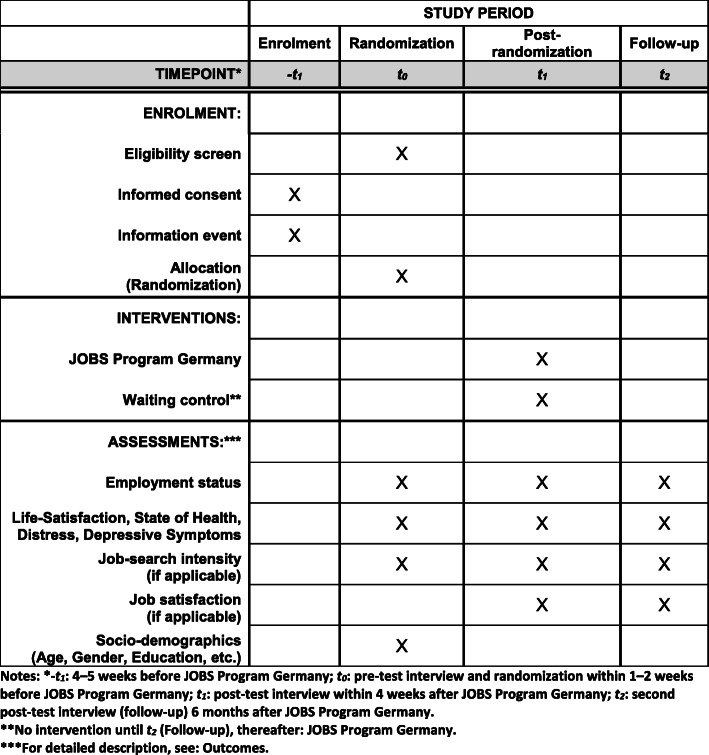
Schedule of enrollment, interventions, and assessments

This confirmatory study is designed as a multi-center, non-blinded, two-armed, parallel-group, randomized controlled trial over 6 months. This design is oriented towards two previous studies from the US and Finland [10, 11]. Participants will be randomly assigned to either an intervention group, who completes JOBS Program Germany right away or a waiting control group, who will not participate in JOBS Program Germany during the trial. The allocation ratio will be 1:1. Primary and secondary outcomes (see below) will be repeatedly assessed at three points in time: (1) immediately before the intervention (*t*_*0*_), (2) immediately after the intervention (*t*_*1*_), and (3) 6 months after the intervention has ended (*t*_*2*_; see: Fig. 1).

### Study setting

The JOBS Program Germany is embedded into the large-scale prevention program “Linking of Employment Promotion and Health Promotion in the Community Setting” in Germany. This is a joint program by the National Association of Statutory Health Insurance Funds, the health insurance companies, the Federal Employment Agency, Jobcenters, the Association of German Counties, and the Association of German Cities. The BZgA coordinates the joint, country-wide activities in approximately 230 locations. Interventions will be conducted in the community-settings of these locations. The data will be collected centralized by the Institute for Social Sciences and Communication (SOKO) on behalf of BZgA and in close coordination with the research team at the University of Kassel. Computer-assisted telephone interviews (CATI) will be conducted based on questionnaires developed by the research team at the University of Kassel. The data will be analyzed exclusively by the research team at the University of Kassel. The sample will comprise adult men and women who are unemployed according to the German Social Code, Book III, and who are willing to participate in the JOBS Program Germany voluntary.

### Intervention

The intervention is based on the JOBS Program that was developed by the Michigan Prevention Research Center [6, 7]. The JOBS Program Germany for the unemployed is conceptualized as a workshop that combines job application training with elements of social learning theory and self-efficacy [8, 9]. More specifically, participants discuss potential problems, difficulties, and setbacks during the job-search process while also developing coping strategies and the skills to overcome these challenges. Trainers provide emotional support by constantly giving positive feedback. In sum, the JOBS Program Germany centers around the following concepts (for a detailed description, see the respective JOBS Manual [5]):

- Job-search skills
- Active teaching/learning methods
- Inoculation against setbacks
- Trainer referent power
- Social support

Accompanied by a team of two trainers, the intervention group completes JOBS Program Germany in small groups of 8 to 15 unemployed people for 5 days in units of 5 h a day over a period of 1 week. Trainings will be completed in the community-settings at each of the participating locations.

Resembling the Finnish implementation of the JOBS Program [11], trainers will partly be recruited from unemployed jobseekers. In each trainer team, one unemployed person will be paired up with a professional trainer, usually an employee at the local Jobcenter, who has some experience in offering regular job application trainings. The training of the trainers is conceptualized in cooperation with the Finnish Institute of Occupational Health and follows a translated version of the original JOBS Program protocol.

### Waiting control group

While the JOBS Program Germany is free of charge for the participants, similar prevention and health promotion programs for the unemployed, according to paragraph 20 of the German Social Code, Book V, are usually associated with participant fees. Because there is no equivalent free-of-charge health promotion program for the unemployed, we have opted for a passive waiting control group that will not participate in JOBS Program Germany and, thus, not receive effective treatment during the six months of the trial. The unemployed in the waiting control group will complete JOBS Program Germany after the trial has ended.

### Procedure

Due to the country-wide implementation of the JOBS Program Germany in the community-settings, the exact dates of enrollment and trainings will vary across the participating locations. Also, at some locations, more than one JOBS Program Germany will be conducted. However, for any given JOBS Program Germany at a given location, the time schedule is fixed.

Figure 1 shows the time schedule for a typical JOBS Program Germany at a given location. At each of the participating locations and for each JOBS Program Germany there will be an information event for the unemployed. This information event will be scheduled and announced (e.g., via flyer, by agents of the Jobcenters) about 4 to 5 weeks before the JOBS Program Germany starts (−*t*_*1*_). During these information events, two trainers, who will later lead the respective JOBS Program Germany, will explain the intervention and provide participants with a written information sheet that summarizes the objectives, procedure, and contents of the program. Trainers will also address any questions from potential participants. Participation is voluntary and free of charge, and a withdrawal is possible at any time without consequences. At the end of the information events, trainers will obtain written informed consent from the unemployed who are willing to participate.

Following the information event, participants who have given informed consent will be contacted and asked to complete an initial CATI during which participants’ eligibility for the trial is assessed. Participants meet the inclusion criteria if they are between 18 and 65 years old and currently unemployed according to the German Social Code, Book III by the time of the interview. Participants who, by the time of the interview, are full-time students or otherwise inactive on the labour market, and those with insufficient command of the German language to complete the CATI are excluded from the trial.

After assessing the baseline measures, all eligible participants will be randomly assigned to an intervention or waiting control group upon completion of the initial CATI (*t*_*0*_). The allocation (stratified by location) will take place automatically with a 1:1 ratio in an alternating fashion. The first participant who successfully completes the CATI will be allocated to the intervention group; the second participant will be allocated to the waiting control group, and so on. Because allocation is carried out in “real-time”, there is no predetermined list. Hence, whether a participant is allocated to the intervention or control group is not known in advance. Moreover, because at any point in time more than one CATI will be conducted simultaneously, interviewers cannot predict the allocation of a given participant prior to the end of the current interview. The information about the group to which participants will be assigned is only revealed to the interviewers by the time the interview is completed.

Upon completion of the CATI, interviewers will invite participants who are assigned to the intervention group to complete JOBS Program Germany, usually within two weeks. Participants who are assigned to the intervention group might immediately decline participation in the program. The respective persons are then assigned to a separate (waiting control) group and their place in the intervention group is filled with another participant who has not yet been allocated to either group. Any such deviation from the randomly allocated intervention will be recorded in the methods reports and in the dataset. Participants in the waiting control group will be told that JOBS Program Germany will start in 6 months Participants who are randomly allocated to the waiting control group cannot participate in JOBS Program Germany, meaning that it is not possible for participants to self-select into the intervention group.

Participants in the waiting control group are not allowed to join JOBS Program Germany during the 6 months of the trial. However, participants in both the intervention and waiting control group will not actively be discouraged or otherwise prevented from participating in any employment promotion measures of the Federal Employment Agency or any prevention or health promotion measures of their choice other than the JOBS Program Germany. Participation in the intervention is voluntary and may be terminated by the participants at any time.

The post-test interview will take place within 4 weeks after the intervention group has completed JOBS Program Germany (*t*_*1*_). Participants of the intervention group and waiting control group will be asked whether they currently participate or have participated in any measure of the Federal Employment Agency or in any health promotion measure. The intervention group is additionally asked to confirm that they have completed JOBS Program Germany. Primary and secondary outcomes are also assessed.

The final CATI will be conducted 6 months after the intervention group has completed JOBS Program Germany (6-months follow-up, *t*_*2*_). Again, participants in both the intervention and the waiting control group will be asked to report participation in any job-search or health promotion related measure. Also, all outcome measures are assessed. Upon completion of the final CATI, the unemployed in the waiting control group will be invited to complete JOBS Program Germany.

Participants in both the intervention group and the waiting control group will receive a 15 Euro voucher for each completed interview. Participants who, nevertheless, decide to discontinue will be asked for their subjective reasons to do so. We will also collect basic socio-demographic characteristics of all participants during the first interview.

The country-wide recruitment is expected to start in April 2021 and continue until August 2021. We expect that a total of approximately 60 JOBS Program Germany will be conducted between April and September 2021. Given the time schedule with a fixed 6-months follow-up, the trial is expected to end in February 2022.

No adverse effects are expected from completing the JOBS Program Germany. International studies on the JOBS Program found either positive effects ([7], e.g. [10, 11, 13, 14, 17]) or no effects, depending on, e.g., the time span between intervention and follow-up or the specific outcome observed. For instance, Reynolds and colleagues reported several positive effects of the Winning New Jobs Programme (adapted from JOBS Program) implemented in two rural communities in Ireland and Northern Ireland [14]. At the 1-year follow-up, participants of the intervention group were more likely to get reemployed, showed an increased inoculation against setbacks, and showed less economic hardship. However, initially positive effects on job search activities, job-seeking efficacy, and job search motivation did not persist until the 1-year follow-up. Because no adverse effects are expected, the unemployed allocated to waiting control group may join JOBS Program Germany to benefit from any positive effects after the trial has ended. Nevertheless, we will record and publish any negative effects that might occur.

### Outcomes

All outcomes and predictor variables will be measured in telephone interviews based on questionnaires developed by the research team at the University of Kassel. Participants (intervention and waiting control group) will be interviewed before the JOBS Program Germany (*t*_*0*_), immediately after the JOBS Program Germany have completed (*t*_*1*_) and 6 months after the JOBS Program Germany have completed (6-months follow-up, *t*_*2*_) (Fig. 1).

#### Primary outcome measures

##### Reintegration into the labor market

Reintegration into the labor market will be assessed using a binary indicator of participants’ current employment status (employed vs. unemployed). For those participants who are employed, we will also assess whether they have a fixed-term or a permanent employment contract as well as their weekly working hours. Any subsided labor market activities (e.g., “Mini-Job”) will be assessed, additionally.

##### Self-esteem and self-efficacy

Participants’ self-esteem is measured with a revised German version of the widely used Rosenberg Self-Esteem Scale [18, 19], for which an internal consistency of Cronbach’s alpha = .84 is reported. Self-efficacy is measured using three short scales for assessing the internal-external control conviction [20], general self-efficacy [21], and generalized self-efficacy expectation [22]. Reliability for the first two scales are reported in terms of McDonald’s omega and lie in the ranges of .53–.73 and .81–.87, respectively. Cronbach’s alpha for the generalized self-efficacy expectation is reported as .74–.94.

##### Health and health-related outcomes with an emphasis on mental health

We assess life satisfaction with a German version of the Satisfaction with Life Scale (SWLS) [23]. A recent study has tested the psychometric properties of the SWLS in a general population survey in Germany [24]. The results confirm that the SWLS is one-dimensional, and indicate measurement invariance across gender and age. Furthermore, satisfaction with life proofed to be associated with fatigue, the mental health component of quality of life, anxiety, dispositional optimism, pessimism, and sleep quality.

We assess participants’ health status by asking for a self-evaluation of their overall state of health (answers on a 5-point Likert-type Item labeled from “very good” to “very bad”). Additionally, we examine whether participants feel restricted in their everyday activities by chronic health problems. Moreover, we use the Patient-Health-Questionnaire 15 (PHQ-15) to assess the severity of somatic symptoms.

Psychological distress is measured with a short, 21-Item version of the depression-anxiety-stress-scale (DASS-21). Psychometric properties of the DASS-21 (e.g., Cronbach’s alpha = .78–.89) have been assessed in Germany [25] and the DASS-21 has been successfully administered via telephone interviews in various countries [26].

Depressive symptoms are measured using the WHO Well-Being Index (WHO-5) and the Patient-Health-Questionnaire 9 (PHQ-9). The WHO-5 consists of five questions, which assess the subjective well-being. A systematic review, including 213 studies, suggests that the WHO-5 is an adequate instrument for screening depressive symptoms in clinical trials [27]. The WHO-5 has also been successfully applied in the German context [28]. The PHQ-9 module consist of 9 items that are specifically designed to assess depression and have been widely used in clinical contexts as well as in research [29]. It has also been shown that result from the self-administered PHQ-9 are comparable to results for the PHQ-9 administered in telephone interviews [30].

#### Secondary outcome measures

The duration of unemployment, job-search intensity, and, if applicable, job satisfaction are collected via telephone interviews at all three occasions (*t*_*0*_, *t*_*1*_, *t*_*2*_, see Fig. 1). We measure job-search specific self-efficacy with a single Likert-type item that assess the likelihood of finding a job. At the baseline (*t*_*0*_), we also assess basic socio-demographic characteristics, such as participants’ age, gender, citizenship, and level of education.

### Sample size

This trial is conceptualized as a confirmatory study. The respective studies in the US and Finland reported small effect sizes (Cohen’s *d*) ranging from about .14 to .32. for self-esteem, self-efficacy, psychological distress, and depressive symptoms [7, 11]. In order to detect effect sizes of .2, which we deem to be of practical relevance, with 85% power and the conventional alpha-level of .05 (two-tailed t-test), we require a sample size of 450 persons per group. This sample size also allows us to detect a 10 percentage points difference in reemployment with 85% power and alpha-level of .05, assuming a reemployment rate of 30% in the waiting control group. To arrive at our target sample size, we aim at recruiting a total of 1500 unemployed, assuming a dropout rate of 60% between the pre-test (*t*_*0*_) and the 6-months follow-up (*t*_*2*_). We further expect about 60% of the unemployed who attend the information event to participate in our study, resulting in an estimated 2500 unemployed who attend an information event. We summarize our assumptions about response rates and panel attrition in Table 1.

**Table 1:** Assumptions about number of participants, response rates, and panel attrition

	Information Event	Allocation/Pre-Test	Post-Test	6-months follow-up
	*-t*_*1*_	*t*_*0*_	*t*_*1*_	*t*_*2*_
Response rate (per cent)		60%	80%	75%
Intervention group (n)	2500	750	600	450
Waiting control group (n)		750	600	450
Total (n)	2500	1500	1200	900

Notes: -*t*_*1*_: 4–5 weeks before JOBS Program Germany; *t*_*0*_: pre-test interview and randomization within 1–2 weeks before JOBS Program Germany; *t*_*1*_: post-test interview within 4 weeks after JOBS Program Germany; *t*_*2*_: second post-test interview (follow-up) 6 months after JOBS Program Germany

### Data collection and management

The first information events for recruiting participants will take place in March 2021 at four locations. While participants in these four locations follow the regular procedure and time schedule, as described above, the collected data will not be included in the (main) study and will not be part of the analyses. Instead, participants in these first four locations are treated as a pilot sample. This pilot sample will comprise approximately 50 participants who will be allocated to intervention and waiting control groups as described above. The main study is expected to start in April 2021. Participants in the pilot-study will complete JOBS Program Germany (if assigned to the intervention group), the post-test interview (*t*_*1*_), and the 6-months follow-up interview (*t*_*2*_), respectively (Fig. 1), about 1 to 2 months prior to participants of the main study. Questionnaires will be adjusted, if needed, within these 1 to 2 months before being administered to participants of the main study.

Questionnaires are developed by the research team at the University of Kassel, and implemented by SOKO in the CATI software Voxco. The implementation includes automated plausibility checks and filters. Interviews will be conducted by experienced interviewers, who will be trained to administer the specific questionnaires. Interviews will, on average, take 30 min to complete. A detailed methods and field report, including a description of quality assurance measures such as training and supervision of the interviewers, and data processing procedures will be compiled.

The Data will be collected and stored at SOKO, and handled in strict compliance with the German Federal Data Protection Act and the European General Data Protection Regulation (DSGVO). Participants’ contact information will be stored separately from the survey data (i.e., participants’ answers). After data collection, each respondent will be assigned a unique identification code, and the survey data (but not the contact information) will be transferred to the research team at the University of Kassel. Data transfer will be conducted via a secure data exchange server. The entire process of data collection, processing, storage, and backup will be documented in a detailed report.

### Statistical methods

Statistical methods will include t-tests (or equivalent tests for proportions) as well as linear and (binary) logistic regression models. We will compare primary and secondary outcomes between the intervention and waiting control groups.

Reintegration into the labor market (employed vs. unemployed) and the type of contract (temporary vs. permanent) will be assessed using t-tests (or equivalent test of proportions) and logistic regression models. In all analyses, we will treat Likert-Type items as continuous variables. For life-satisfaction, depressive symptoms, and psychological distress, we will first construct (additive) indices. We will then test mean differences in the respective indices between intervention and waiting control groups using t-tests. In multivariate analyses, we will use linear regression models to adjust for baseline-measures of the respective outcomes. Additional models will also adjust for socio-demographic characteristics and indicators of labor market biography, such as the duration of unemployment. We proceed in a similar fashion to analyze self-esteem, self-efficacy and our secondary outcomes job satisfaction and job-search intensity.

To test for potential effect modifications, we include interaction terms between our treatment-indicator and the respective moderators (job-search intensity, duration of unemployment) in the respective regression models.

All multivariate analyses will include controls for socio-demographic variables and (un-)employment history. We will repeat our main analyses stratified by gender. All analyses will be carried out according to the  “intention-to-treat” principle. Participants will be treated as belonging to the group to which they were originally (randomly) allocated. We tolerate low proportions of missing values of up to 5%, and perform listwise-deletion (complete-case analyses). If the proportion of missing values exceeds the predefined threshold of 5%, we will treat missing values by means of multiple imputation [31–33].

### Interim analyses

Interim analyses will be carried out using the statistical methods described above immediately after JOBS Program Germany have been completed (Fig. 1, *t*_*1*_). The research team of Kassel University is obliged to provide research reports to the funder on a yearly basis. Thus, interim results will be reported to the funder. We will also report interim results to the BZgA. The trial will not be modified or terminated before the scheduled 6 months regardless of the results of any interim analyses.

### Frequency and plans for auditing trial conduct

The research team at the University of Kassel will hold meetings with BZgA, National Association of Statutory Health Insurance Funds, and SOKO on a monthly basis or more frequently if required (e.g., during the time of recruitment). In these meetings, any organisational aspects, including but not limited to recruitment of locations and participants, participation rates in JOBS Program Germany, and overall time schedule are monitored and discussed. Corrective actions to preserve the integrity of the study will be taken if deemed appropriate.

## Dissemination plans

Results will be published via multiple channels. The funding agency, the Federal Ministry of Education and Research, will be informed on a regular basis, at least once a year, about the progress of the project and also about the interim and final results.

Furthermore, the cooperating partners, above all the BZgA, will be informed about the study results by the research team at the University of Kassel. The BZgA covers a wide range of media, including regular newsletters, which can provide information about the JOBS Program Germany and the respective study results.

The research team at the University of Kassel will report all results of the confirmatory evaluation study independently from the founding party or any cooperating partners, and regardless of strength and direction (positive, negative, null-results) of the effects. This reporting will take place in the context of university teaching, at national and international labor- and public health-related conferences, and in the form of publications in international peer-reviewed journals. Authorship eligibility will be based on the recommendations of the international committee for medical journals editors (ICMJE).

The various publication channels ensure that no result remains unknown and that the dissemination will be tailored to the different target groups. In addition to the publication of the results in the international scientific community, it is ensured that university students, the unemployed in Germany, health organizations and health insurance companies, institutions of employment promotion and their practitioners, as well as political decision-makers will be fully informed about the study results. This ensures low-threshold and transparent accessibility of the results and prevents publication bias.

### Plans to give access to the full protocol, participant level-data and statistical code

Access to the full study protocol is regularly granted to the funder and, upon request and after a formal agreement on confidentiality, to interested scientists. Because informed consent, sought from the participants, does not include general release of the data, we will not publish the participant level datasets. Statistical code will be made available via appropriate data repositories.

### Plans for communicating important protocol amendments to relevant parties

Substantive modifications to the study protocol, including but not limited to changes of the study objectives, the eligibility criteria, the sample sizes, the allocation, the outcomes, or the statistical analyses will be discussed with all cooperation partners. Any such changes will be documented and published as amendments to this study protocol. Amendments shall also be made to the trial registry entry in the DRKS. Specific attention must be drawn to the course of the SARS-CoV-2/COVID-19 pandemic that might impair participant recruitment or may hamper the education of potential trainers.

## Discussion

Compared to the employed, the risk of morbidity is significantly higher for unemployed persons in Germany. Furthermore, the prevention and health promotion hardly reach the unemployed. The JOBS Program is a health promotion program that specifically targets the unemployed. It combines job application training with elements of social learning theory and self-efficacy to improve the reintegration into the labor market and (mental) health.

In this confirmatory study, we assess whether the positive effects of the JOBS Program in the United States [7], Finland [11] and the Netherlands [17] can be replicated in the German context. The greatest strength of our study design is that we implement a randomized controlled trial in a large-scale, countrywide field experiment, thus, maximizing both the internal and external validity of our results. However, even this “real-life” setting has certain limitations. Most importantly, our trial will be conducted during the current SARS-CoV-2/COVID-19 pandemic that will probably affect, directly or indirectly, most of our outcomes, such as reintegration into the labor market and mental health. Assuming that the pandemic will have comparable effects for both the intervention group and the waiting control group, we do not expect large impacts on the internal validity of our results. However, our time schedule might be delayed by future government measures (“shutdown”) that temporarily hamper implementation of JOBS Program Germany.

Despite the discussed limitations, our study potentially provides the basis for a valuable addition to the German employment promotion and social security system. If the positive effects on reemployment and (mental) health can be replicated in the German context, the JOBS Program Germany could be implemented on a regular, long-term basis to benefit the unemployed. Since the administrative infrastructure is already in place and the relevant agencies are already involved, implementation of the JOBS Program Germany could perspectively be extended.

## Trial status

This is version 1 of the study protocol, submitted in December, 2020. Recruitment is expected to start in March, 2021. Recruitment will approximately be completed by the end of September 2021.

## Data Availability

Not applicable.

## Abbreviations

BZgA: Federal Centre of Health Education (Bundeszentrale für gesundheitliche Aufklärung)
CATI: Computer-assisted telephone interview
DASS-21: Depression-Anxiety-Stress-Scale
PHQ-9: Patient-Health Questionnaire 9
SWLS: Satisfaction with life scale
WHO-5: World Health Organization well-being index
